# Intraductal papilloma of breast with carcinoma *in situ* and lymph node tuberculosis: a case report

**DOI:** 10.3389/fonc.2025.1640302

**Published:** 2025-10-29

**Authors:** Lu Zhao, Li Wu, Shuai Luo, Yao Li, Jinjing Wang

**Affiliations:** Department of Pathology, Affiliated Hospital of Zunyi Medical University, Zunyi, Guizhou, China

**Keywords:** intraductal papilloma, ductal carcinoma *in situ*, breast, tuberculous lymphadenitis, pathology, treatment

## Abstract

**Background:**

The simultaneous occurrence of intraductal papilloma and malignant breast lesions is rare, as is the presence of ductal carcinoma *in situ* (DCIS) alongside axillary tuberculous lymphadenitis. In DCIS cases, axillary lymphadenopathy should initially raise concerns about metastasis; however, other conditions, such as granulomatous diseases or alternative malignancies, may present with similar clinical features. Given the patient’s extended residence in a tuberculosis-endemic region and history of pulmonary tuberculosis, determining the etiology of axillary lymphadenopathy is imperative. Thus, an accurate pathological diagnosis, exclusion of metastatic breast cancer, and precise staging are essential for effective management.

**Case presentation:**

A 49-year-old female with a decade-long history of pulmonary tuberculosis presented with a right breast mass, first noticed 10 days prior. Color Doppler ultrasound identified a hypoechoic nodule in the right breast, and breast MRI, both with and without contrast, revealed multiple regions of abnormal enhancement in the right breast, along with enlarged axillary lymph nodes. Tissue biopsy confirmed intraductal papilloma with malignant transformation in some areas (low-grade DCIS). Axillary lymph node biopsy was positive for tuberculosis. The patient underwent right mastectomy, and follow-up after one month showed no recurrence.

**Conclusion:**

Tuberculosis remains prevalent in developing countries, and this patient’s history of pulmonary tuberculosis spans over 10 years. The co-occurrence of breast cancer and axillary tuberculous lymphadenitis is unusual. A prompt and accurate diagnosis of breast cancer is vital, involving preoperative imaging, biopsy, and pathological assessment during and after surgery. Once diagnosed, a treatment plan tailored to the characteristics of carcinoma *in situ* should be implemented, alongside concurrent treatment for tuberculosis, to improve the patient’s prognosis.

## Background

Intraductal papilloma, accounting for 5.3% of benign breast conditions, is a non-malignant breast tumor (1). Ductal carcinoma *in situ* (DCIS) is characterized by the non-invasive proliferation of neoplastic glandular epithelial cells confined within the mammary ductal-lobular system. These cells exhibit adhesive properties and demonstrate varying nuclear grades, leading to diverse tissue structures. The substantial heterogeneity of DCIS contributes to variability in clinical presentation, histopathological features, biomarker expression, and genetic and molecular profiles, thereby emphasizing the necessity for early detection and timely surgical intervention in breast cancer management. DCIS is categorized into low, intermediate, and high nuclear grades (2). The simultaneous presence of intraductal papilloma and malignant breast lesions is rare (3). The co-occurrence of breast cancer and tuberculosis in axillary lymph nodes is also infrequent in clinical practice (4). Consequently, when axillary lymphadenopathy is detected in a breast cancer patient, metastasis is generally suspected as the primary diagnosis, although alternative conditions, such as granulomatous diseases or other malignancies, may present with similar clinical manifestations (5). Considering the patient’s extended residence in a tuberculosis-endemic region and history of pulmonary tuberculosis, differentiating the underlying cause of axillary lymphadenopathy becomes imperative. This scenario poses a considerable diagnostic and therapeutic challenge for clinicians.

## Case presentation

A 49-year-old female with a 10-year history of pulmonary tuberculosis presented with a right breast mass persisting for more than 10 days. A physical examination conducted two weeks earlier identified a right breast mass of indeterminate size, non-tender, with no nipple discharge or bleeding. No associated symptoms, including chills, fever, fatigue, anorexia, low-grade fever, night sweats, chest tightness, chest pain, or dyspnea, were reported.

Upon physical examination, a firm, patchy thickening was palpated at the 9 o’clock position of the right breast. The mass was mobile, non-tender, well-circumscribed, and not adhered to the chest wall or skin. No nipple discharge was observed, and the left breast was unremarkable. Enlarged lymph nodes were palpable in the right axilla, while no abnormal nodes were detected in the left axilla or the supra- and infraclavicular regions.

Auxiliary examination: Breast color Doppler ultrasound revealed hypoechoic nodules in the right breast, with intraductal lesions suspected. Abnormal lymph nodes were also noted in both axillary regions.

**Figure 1:**
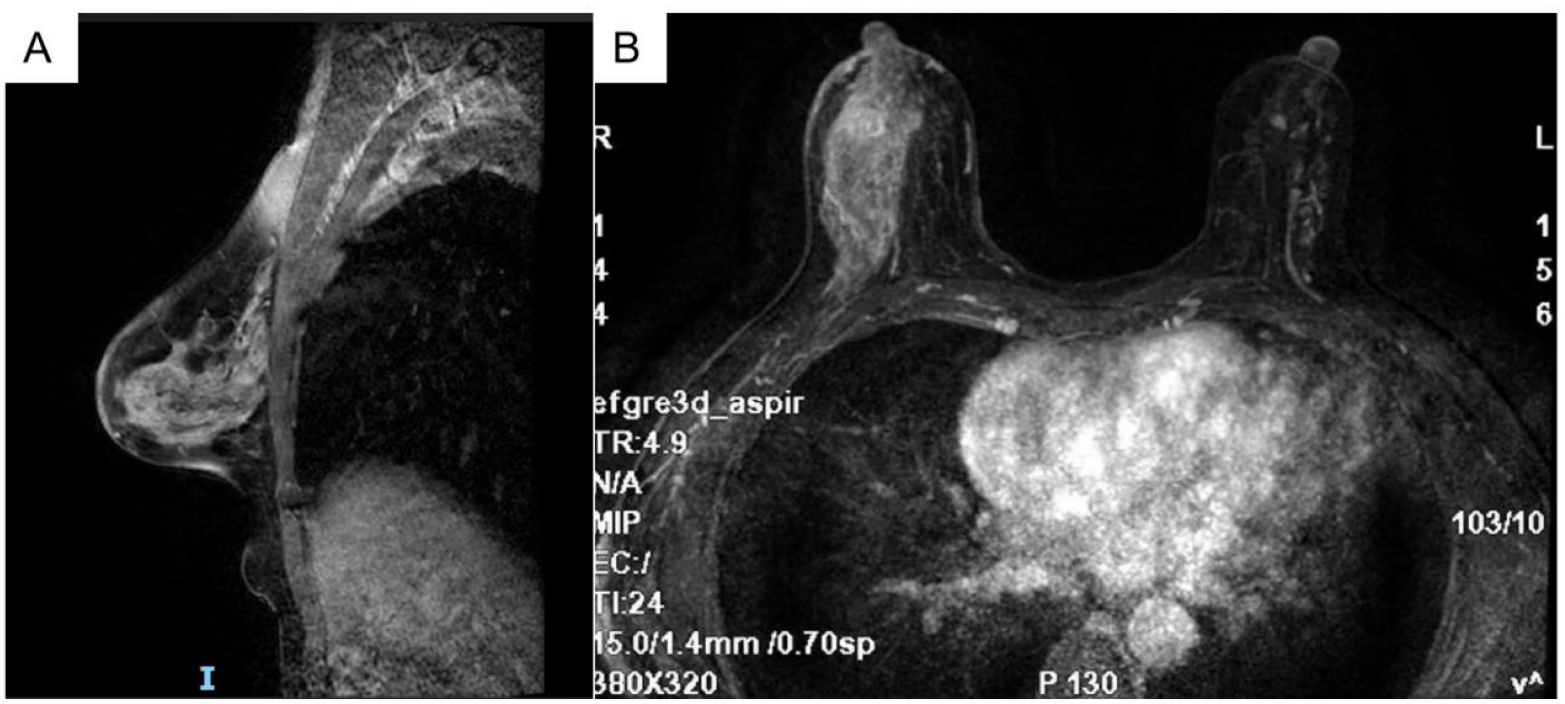
Bilateral breast c-shaped images with low background enhancement and multiple regional abnormal enhancement areas in the right lateral quadrant of the breast [**(A)** Sagittal section **(B)** Transverse section].

The breast MRI (**Figure 1**), both with and without contrast administration, identified C-type bilateral breasts with low background enhancement. The outer quadrant of the right breast exhibited multiple areas of abnormal enhancement, nearly including the entire region. Following contrast injection, enhancement appeared markedly irregular, with a significant increase in vascular shadows both within and around the affected areas. The time-signal intensity curve conformed to type II, while diffusion was restricted (1.1). Enlarged lymph nodes in the right axilla, accompanied by surrounding edema and high signal diffusion, indicated multiple regions of abnormal enhancement in the right breast and enlarged axillary lymph nodes.

Given the indeterminate nature of the breast mass, a “right breast mass hollow needle puncture biopsy under color Doppler ultrasound guidance + axillary lymph node puncture biopsy” was performed. Pathological examination, including morphological and immunohistochemical analysis, confirmed the presence of a malignant tumor, most likely low-grade DCIS.

The pathological findings necessitated local resection of the right breast lesion and regional lymph node dissection, with tissue samples submitted for further microscopic evaluation.

**Figure 2:**
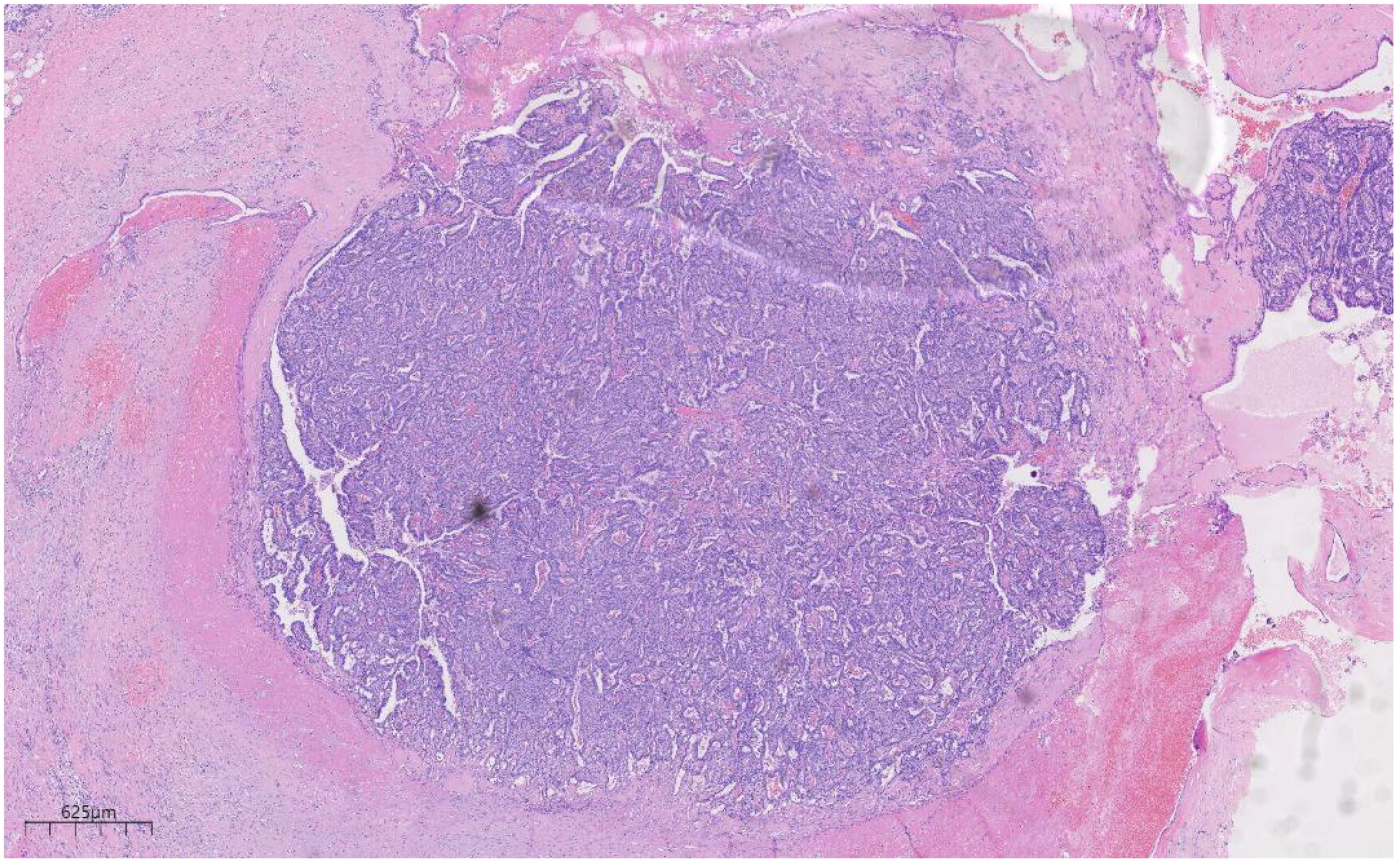
Low magnification shows papillary hyperplasia in the breast duct with clear margins. H&E ×15.7.

**Figure 3:**
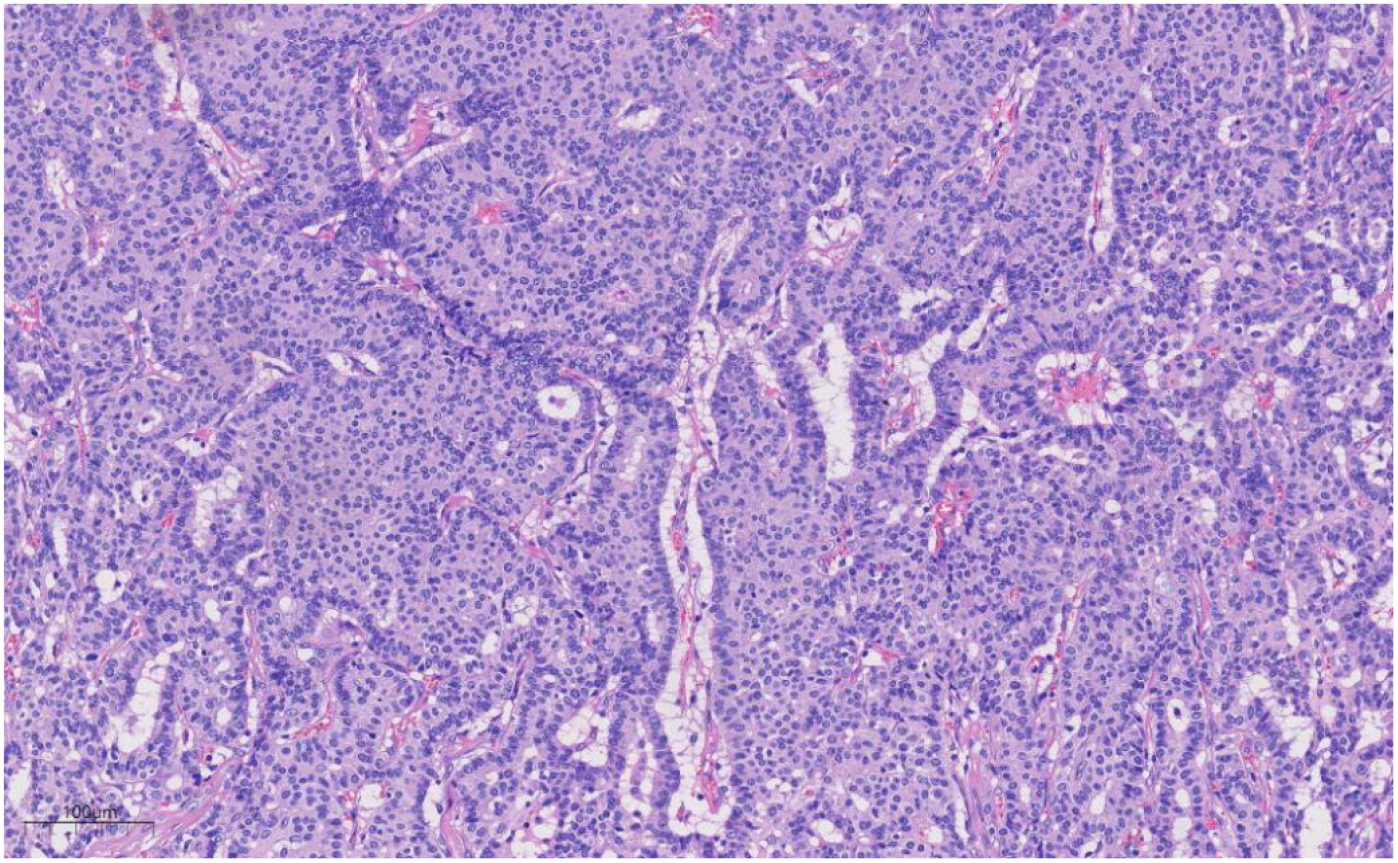
Under medium magnification, low-grade DCIS was papillary and solidly arranged, with oval to round nuclei of uniform size and often arranged in a very high direction. H&E ×100.

**Figure 4:**
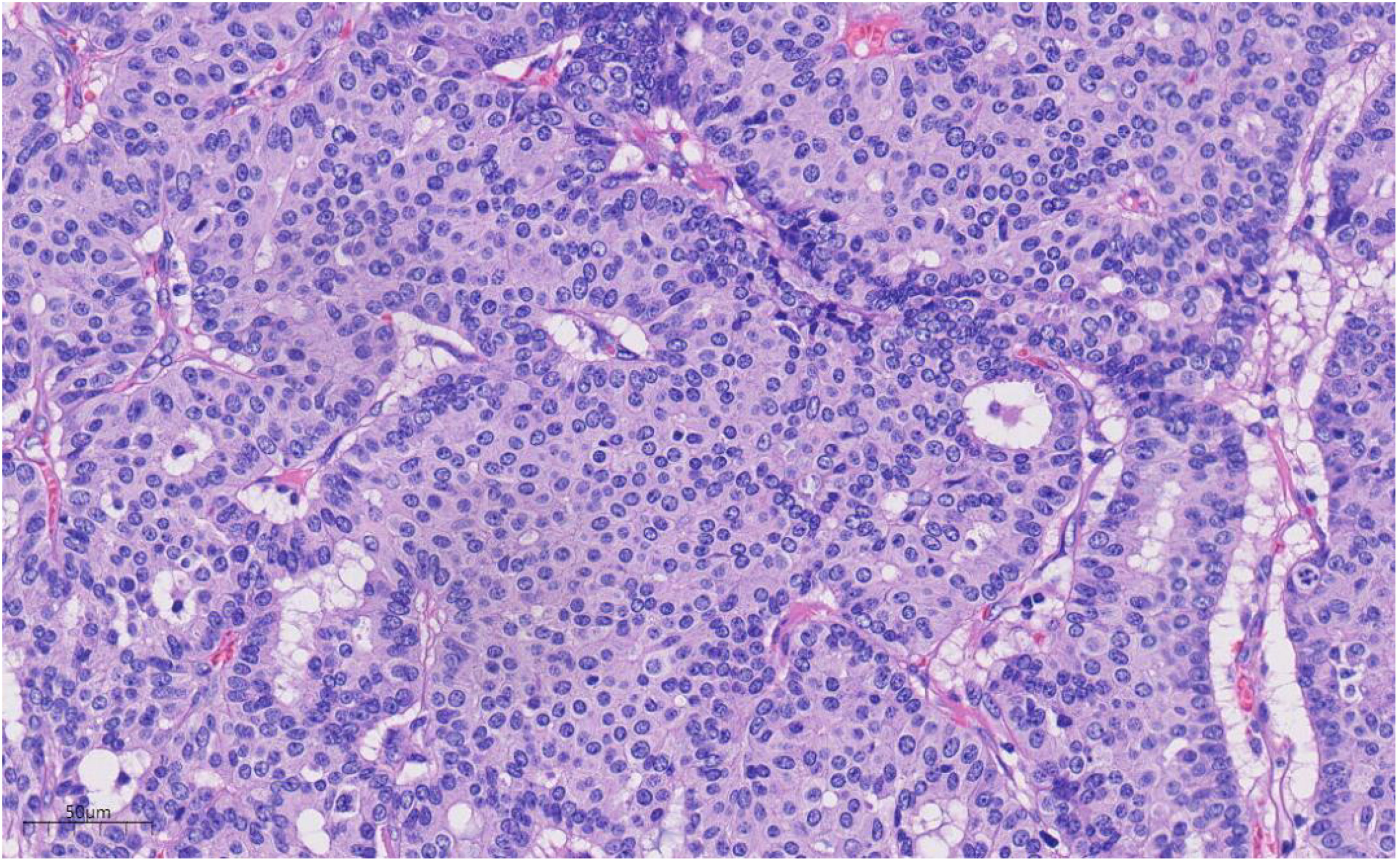
Under high magnification, the nuclear membrane of the nucleus is smooth, the chromatin is uniform, and the chromatin is dust-like to fine granular. The nucleolus is not obvious, and the nuclear division is rare. H&E ×270.

**Figure 5:**
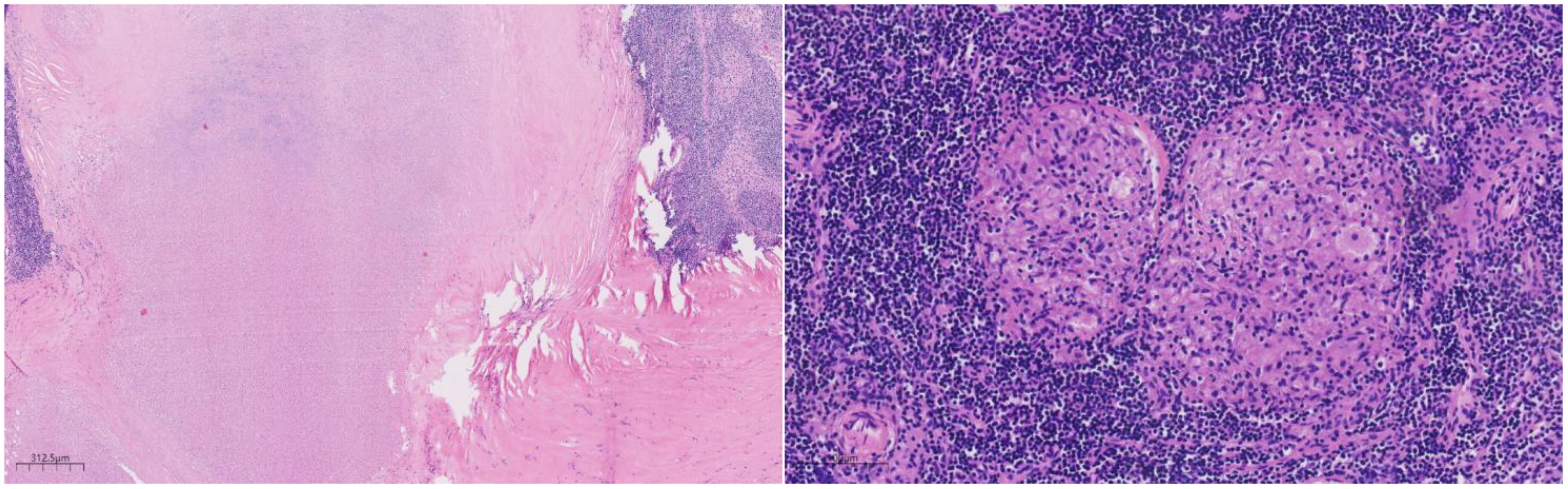
Under the microscope, a large area of necrosis can be seen in the lymph node, surrounded by fibrous tissue or granuloma. H&E ×27.

Histologically (**Figures 2**–**4**), the benign intraductal papilloma exhibited a papillary structure comprising two cellular layers: an inner epithelial layer and an outer basal myoepithelial layer, the latter extending continuously along the papillary axis and duct wall (**Figures 2**-**4**). The lesion contained low-grade DCIS, which demonstrated a papillary structure with solid or window-like formations. Nuclei were predominantly arranged in a polar orientation, slightly enlarged, oval to round in shape, uniform in size, and without overlap. Nuclear membranes were smooth, chromatin appeared uniform, varying from dusty to finely granular, and nucleoli were inconspicuous, with mitotic figures rarely seen. Notable areas of necrosis were observed in the axillary lymph nodes, surrounded by fibrous tissue or granulomas (**Figure 5**).

**Figure 6:**
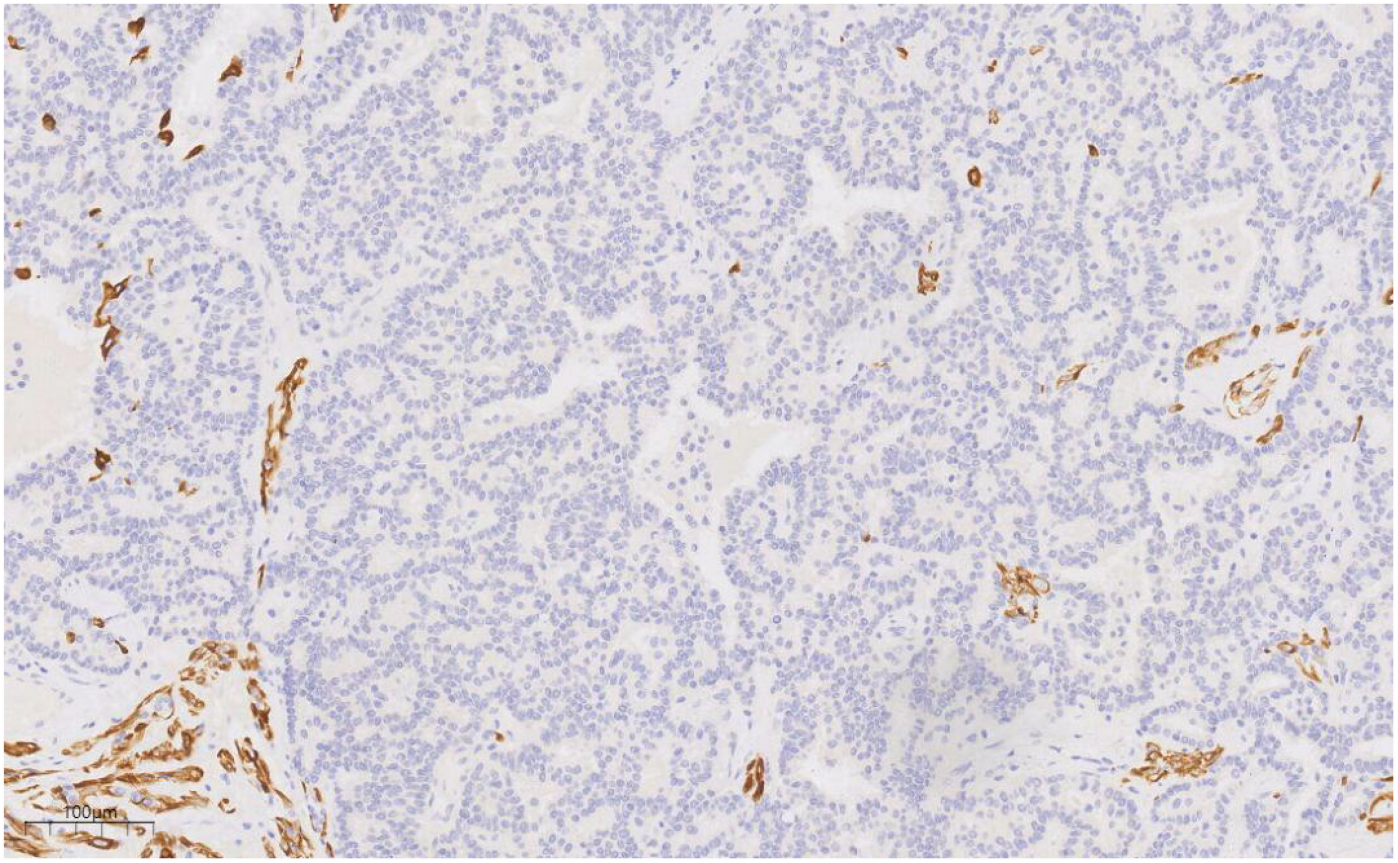
CK5/6 labeling results suggest the presence of myoepithelial cells around the duct, and the absence of myoepithelial cells in some areas of the proliferative duct epithelium. EnVision, ×100.

**Figure 7:**
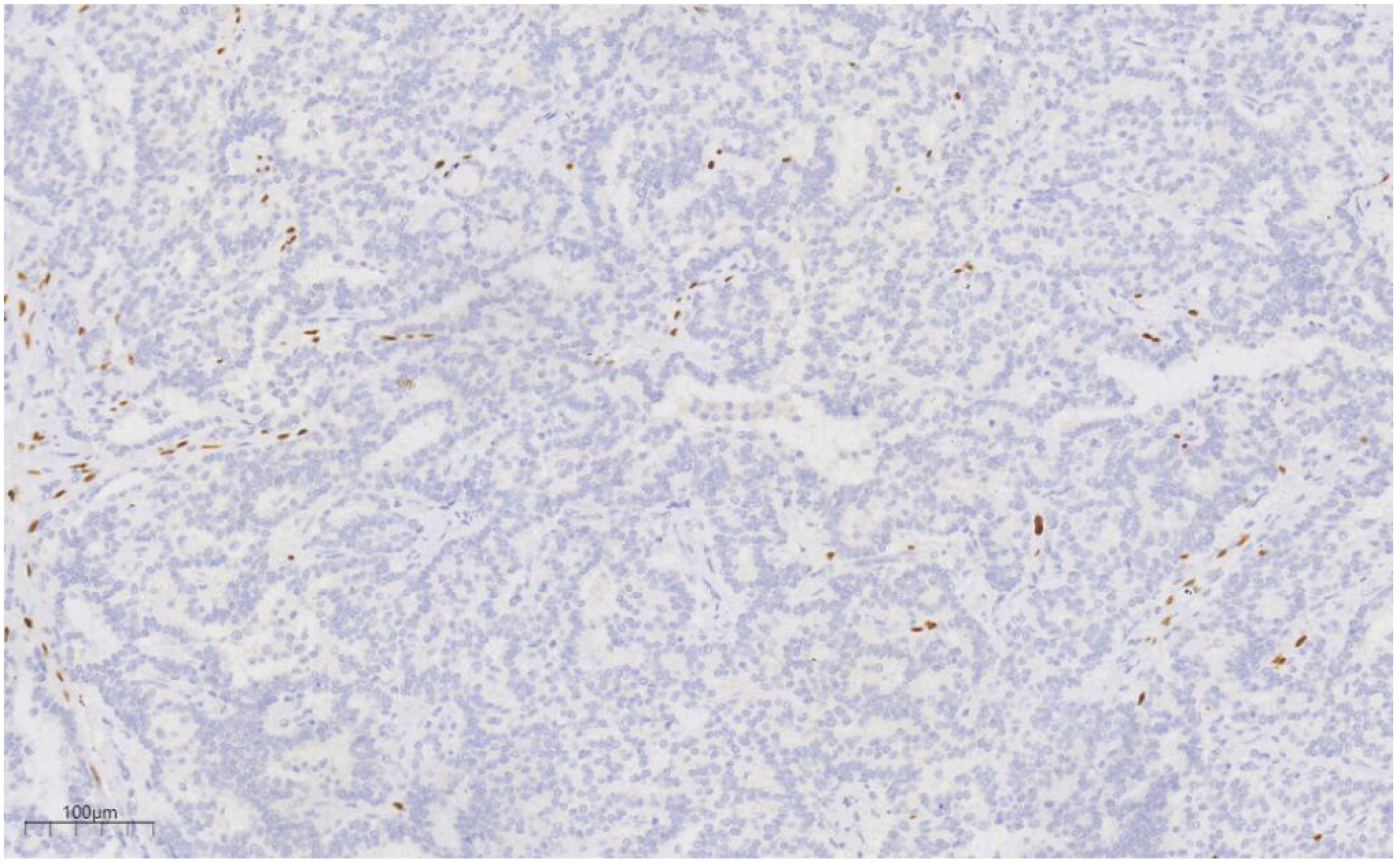
p63 labeling results suggest the presence of myoepithelial cells around the duct, and the absence of myoepithelial cells in some areas of the proliferative duct epithelium. EnVision, ×100.

Immunohistochemical analysis revealed strong CK (+++) expression and focal CK5/6 (+) expression in the hyperplastic ductal epithelium. Co-expression of CK5/6 (**Figure 6**) and p63 (**Figure 7**) confirmed the presence of myoepithelial cells encircling the ducts, though some hyperplastic regions exhibited loss of myoepithelial cells. ER and PR were moderately positive, with expression levels exceeding 90%, HER2 was scored 0, and the Ki-67 labeling index was approximately 20%.

Special stains (AB-PAS, PAS) showed no evidence of fungal elements, while acid-fast staining was negative for acid-fast bacilli.

Molecular pathology identified *Mycobacterium tuberculosis* DNA via PCR.

The pathological biopsy, in alignment with the consensus diagnostic criteria (2), confirmed the diagnosis of (right) intraductal papilloma accompanied by low-grade DCIS. Immunohistochemical analysis further supported the morphological findings. Considering the patient’s history of pulmonary tuberculosis and molecular pathological results, the diagnosis of right axillary lymphadenectasis was established as tuberculous lymphadenitis.

Subsequently, the patient underwent a right unilateral mastectomy, with tissue samples sent for histopathological evaluation. Gross examination revealed a single specimen with a fusiform skin flap measuring 190 mm × 130 mm × 25 mm; the skin flap itself was 130 mm × 75 mm, and the nipple diameter was 13 mm. No nipple retraction or orange peel-like changes were noted. A 50 mm incision was positioned at the 9 o’clock axis, 5 mm from the nipple. Beneath this incision, an 8 mm-deep cavity was observed, measuring 40 mm × 40 mm × 10 mm, with no discernible induration or irregularities within the cavity. Microscopic examination revealed multilayered ductal epithelial proliferation, marked by cellular pleomorphism, structural disorganization, and loss of polarity.

At the three-month postoperative follow-up, the patient demonstrated satisfactory recovery, with no signs of recurrence.

## Discussion

Intraductal papilloma, a benign breast neoplasm, may or may not present as a palpable mass (1). The concurrent occurrence of malignant breast lesions is rare (3). Tuberculosis continues to represent a major global public health challenge, especially in developing regions. The coexistence of breast carcinoma and axillary tuberculous lymphadenitis is an uncommon finding. Considering the patient’s prolonged residence in a tuberculosis-endemic area and the documented history of pulmonary tuberculosis, axillary lymphadenopathy does not rule out tuberculous lymphadenitis. Histopathological biopsy, with its higher diagnostic precision, surpasses other diagnostic techniques and is crucial in guiding therapeutic decisions and predicting prognosis (4, 5). The presence of both breast cancer and axillary tuberculous lymphadenitis complicates the formulation of effective treatment strategies and attainment of optimal outcomes. The subsequent discussion examines the clinicopathological characteristics, differential diagnosis, and therapeutic approaches to improve the clinical recognition and management of this rare clinical presentation.

### Clinical characteristics

DCIS generally manifests as a firm, poorly defined, painless mass with limited mobility. Axillary lymphadenopathy may be present, often accompanied by mild tenderness, while systemic symptoms, such as low-grade fever or night sweats, are typically absent. Concurrent breast cancer and tuberculous lymphadenitis are rarely reported (4). The patient in this case, a 49-year-old woman, presented with a painless mass in the right breast, devoid of nipple discharge or skin changes. Although axillary lymphadenopathy in breast cancer often indicates metastasis, this case revealed an alternative cause.

### Imaging studies

Breast ultrasound demonstrates greater sensitivity in detecting papillomas, often showing ductal dilatation and solid intraductal echogenic nodules (6). In comparison, MRI proves more effective in identifying DCIS, which typically appears as segmental or ductal mass/bunchiness, non-mass enhancement within ductal structures (7). In tuberculous lymphadenitis, early-stage nodal hyperplasia may be associated with caseous necrosis on imaging (8). In the current case, imaging studies did not provide a conclusive diagnosis. Given the limitations of radiological techniques, histopathological biopsy is critical for accurate diagnosis.

### Pathological characteristics

#### Microscopic findings

Papilloma-like lesions commonly present a papillary structure with a fibrovascular core. In benign intraductal papilloma, the papillary structures are lined by a bilayered epithelium, comprising an inner luminal epithelial layer and an outer myoepithelial layer. Myoepithelial cells are evenly distributed along the papillary axis and ductal walls. In this case, the low-grade DCIS component exhibited papillary, solid, and fenestrated growth patterns. Nuclei were arranged in a preserved polarity, mildly enlarged, oval to round, non-overlapping, and uniform in size. Nuclear membranes appeared smooth, with chromatin ranging from dusty to finely granular and inconspicuous nucleoli; mitotic activity was rare. Apocrine differentiation was focally evident, with cuboidal to columnar cells forming localized clusters. Occasionally, the papillary axes were lined by a single layer of columnar apocrine cells. Histopathological evaluation effectively distinguished benign papilloma from intraductal carcinoma, facilitating precise diagnostic classification. Furthermore, axillary lymph node sections demonstrated characteristics consistent with tuberculosis, including extensive necrosis bordered by fibrous tissue or granulomas, differing from the cytological profile of metastatic carcinoma.

#### Immunophenotype

Immunohistochemistry is essential for the characterization of papillary lesions. In this case, myoepithelial markers, including p63 and basal cytokeratins (CK5/6), were consistently expressed along the majority of the papillary axes and ductal periphery, confirming the presence of an intact myoepithelial layer surrounding the papillary structure. In contrast, the epithelial component within the DCIS foci exhibited a uniform, monoclonal profile: tumor cells demonstrated strong and diffuse ER positivity, coupled with the complete absence of high molecular weight keratins (e.g., CK5/6). Immunohistochemistry also plays a crucial role in assessing invasion and refining the differential diagnosis. The loss of myoepithelial marker expression (e.g., p63, SMMHC) in the fibrovascular core or periphery of the lesion generally suggests papillary carcinoma over benign papilloma (9, 10). In this case, although focal attenuation of myoepithelial staining was observed within portions of the papillary axis, a continuous p63-positive myoepithelial layer encircled the ducts, supporting an *in situ* process. The immunophenotypic findings, in combination with the morphological features, confirm a predominantly benign papilloma with focal low-grade DCIS.

#### Molecular genetics

Recent genomic studies have revealed PIK3CA mutations in approximately 69% of papilloma cases, typically in the absence of significant copy number alterations. However, as these lesions progress to concurrent DCIS or invasive carcinoma, malignant clones often lack PIK3CA driver mutations, instead displaying complex copy number alterations across multiple chromosomal regions. These findings suggest that papilloma may serve as a precursor lesion in breast cancer, where early oncogenic events, such as PIK3CA mutations, initiate clonal expansion, followed by the accumulation of genomic instability as malignancy advances (11). PCR has been established as a sensitive and rapid technique for detecting *Mycobacterium tuberculosis* (12). In this case, granulomatous inflammation and extensive necrosis were observed in the lymph nodes, indicating tuberculosis histologically; this diagnosis was confirmed by a positive tuberculosis PCR, thereby corroborating the identification of tuberculous lymphadenitis.

### Differential diagnosis

(1) Encapsulated papillary carcinoma primarily affects postmenopausal women and is characterized by a well-defined fibrous capsule that separates the lesion from surrounding breast tissue. Neoplastic cells in low-grade DCIS remain confined to the ductal system without breaching the basement membrane. Immunohistochemical profiling is essential for differentiation; in low-grade DCIS, ER and PR expression typically appears clonal and diffusely distributed, which aids in distinguishing it from encapsulated papillary carcinoma.

(2) Lymph node metastatic carcinoma is initially considered due to the presence of axillary lymphadenopathy in this patient with confirmed DCIS. However, given the patient’s prolonged residence in a tuberculosis-endemic area and a history of pulmonary tuberculosis spanning over a decade, additional evaluation is warranted. Histopathological examination reveals no features indicative of metastasis, and molecular testing for TB-PCR is positive, ruling out metastatic carcinoma and confirming tuberculous lymphadenitis.

### Treatment and prognosis

DCIS presents a risk of progression to invasive ductal carcinoma, necessitating prompt surgical intervention, which is typically associated with favorable outcomes (13). In cases involving both breast cancer and axillary tuberculous lymphadenitis, a combined approach of surgical resection and standardized anti-tuberculosis therapy is essential (14). The patient underwent unilateral right mastectomy with regional lymph node resection). Although the patient had a history of tuberculosis for more than 10 years, The patient received adjuvant chemotherapy and routine anti-tuberculosis treatment after surgery.The identification of tuberculous lymphadenitis in this case highlights the diagnostic difficulty posed by chronic inflammatory conditions during breast cancer staging. While the coexistence of breast cancer and tuberculosis is rare, heightened clinical awareness is crucial in regions with a high prevalence of tuberculosis. This case illustrates the diagnostic and therapeutic challenges stemming from the simultaneous presence of malignancy and tuberculosis, emphasizing the importance of multidisciplinary collaboration to ensure comprehensive management—addressing the carcinoma while concurrently managing infection-related complications.

## Conclusion

This study describes a rare case of concurrent breast cancer and tuberculous lymphadenitis. When axillary lymphadenopathy is present alongside breast cancer, metastatic disease often dominates the differential diagnosis; however, it is essential to consider the clinical history and, when appropriate, incorporate additional diagnostic methods such as special staining and molecular assays to rule out tuberculosis and avoid unnecessary treatments. This case highlights the importance of accurate diagnostic assessment and a multidisciplinary approach to enhance clinical outcomes.

## Data Availability

The original contributions presented in the study are included in the article/supplementary material. Further inquiries can be directed to the corresponding author.
